# Long-term outcome after intensive care for COVID-19: differences between men and women—a nationwide cohort study

**DOI:** 10.1186/s13054-021-03511-x

**Published:** 2021-02-25

**Authors:** Erik Zettersten, Lars Engerström, Max Bell, Gabriella Jäderling, Johan Mårtensson, Linda Block, Emma Larsson

**Affiliations:** 1 Department of Perioperative Medicine and Intensive Care, Karolinska University Hospital, 171 76 Stockholm, Sweden; 2 Department of Physiology and Pharmacology, Karolinska Institutet, Stockholm, Sweden; 3 Department of Cardiothoracic Surgery, Anesthesia and Intensive Care, Linköping University Hospital, Linköping, Sweden; 4 Department of Anesthesia and Intensive Care, Vrinnevi Hospital, Norrköping, Sweden; 5 Department of Medical and Health Sciences, Linköping University, Linköping, Sweden; 6 The Swedish Intensive Care Registry, Karlstad, Sweden; 7 Department of Anesthesiology and Intensive Care, Institution of Clinical Sciences, Sahlgrenska Academy, University of Gothenburg, Gothenburg, Sweden; 8 Department of Anesthesiology and Intensive Care, Sahlgrenska University Hospital, Gothenburg, Sweden

**Keywords:** COVID-19, Intensive care, Long-term outcome, Gender

## Abstract

**Background:**

Questions remain about long-term outcome for COVID-19 patients in general, and differences between men and women in particular given the fact that men seem to suffer a more dramatic course of the disease. We therefore analysed outcome beyond 90 days in ICU patients with COVID-19, with special focus on differences between men and women.

**Methods:**

We identified all patient ≥ 18 years with COVID-19 admitted between March 6 and June 30, 2020, in the Swedish Intensive Care Registry. Patients were followed until death or study end-point October 22, 2020. Association with patient sex and mortality, in addition to clinical variables, was estimated using Cox regression. We also performed a logistic regression model estimating factors associated with 90-day mortality.

**Results:**

In total, 2354 patients with COVID-19 were included. Four patients were still in the ICU at study end-point. Median follow-up time was 183 days. Mortality at 90-days was 26.9%, 23.4% in women and 28.2% in men. After 90 days until end of follow-up, only 11 deaths occurred. On multivariable Cox regression analysis, male sex (HR 1.28, 95% CI 1.06–1.54) remained significantly associated with mortality even after adjustments. Additionally, age, COPD/asthma, immune deficiency, malignancy, SAPS3 and admission month were associated with mortality. The logistic regression model of 90-day mortality showed almost identical results.

**Conclusions:**

In this nationwide study of ICU patients with COVID-19, men were at higher risk of poor long-term outcome compared to their female counterparts. The underlying mechanisms for these differences are not fully understood and warrant further studies.

**Supplementary Information:**

The online version contains supplementary material available at 10.1186/s13054-021-03511-x.

## Introduction

We have not yet seen the end of the Coronavirus disease (COVID-19) pandemic. As of November 17, 2020, there were more than 55 million global cases and more than one million people have died [1]. In late 2020, many countries reported anew increasing number of patients with COVID-19, leading to reintroduction of restrictions including country- or region-wide lockdowns. The emergence of COVID-19 has induced an enormous global need for intensive care services, leading to rapid expansion of intensive care unit (ICU) resources. Understanding of outcomes in COVID-19 patents admitted to ICU is not complete as the current literature to a large extent is based on small patient cohorts and a large variation in follow-up intervals [2, 3]. In addition, many studies are hampered by the fact that many patients still remain in ICU at study endpoint as excluding patients still in ICU when calculating mortality most likely introduces a skewness in the results.

It is by now apparent that men are suffering a more dramatic course of COVID-19 compared to women, and the underlying explanations for these are insufficiently understood. The number of COVID-19 cases appears to be equally distributed between men and women [4], but there are more men than women hospitalized following COVID-19 and more men are treated in the ICU [5–7]. Questions remain about associations of sex with outcome beyond 30 days after ICU admission, after adjusting for age, comorbidities and other clinically relevant confounders. Analysis of comprehensive population data including detailed baseline characteristics, clinical course and minimal loss to follow-up are needed in order to increase clinicians’ knowledge of patient groups at high risk for poor outcome after intensive care treatment. In this nationwide cohort study, we therefore aimed to analyse long-term outcome beyond 90 days in critically ill patients with COVID-19, with special focus on differences between men and women. We also present data on demographics, baseline comorbidities and process of care.

## Methods

This study was approved by the Swedish Ethical Review Authority (approval number 2020-01477). The study adhered to the STROBE (Strengthening the Reporting of Observational Studies in Epidemiology) guidelines for cohort studies [8]. All research was conducted in accordance with national guidelines and regulations.

### Study design and population

Intensive care, as all public health care, is tax-funded and available for all citizens in Sweden regardless of private health insurances. In this nationwide cohort study, we identified all patient ≥ 18 years of age with confirmed SARS-CoV-2 by polymerase chain reaction admitted between March 6 and June 30, 2020, in the Swedish Intensive Care Registry (SIR). We excluded SARS-CoV-2-RNA positive patients with other reason for admission than COVID-19 and patients with temporary or invalid Swedish personal identification number. Patients were followed until death or study end-point October 22, 2020, whichever came first. All 83 ICUs in Sweden are members of SIR. During the inclusion period, 73 of the 83 Swedish ICUs admitted patients with COVID-19. SIR is operative collecting individual patient data within the legal framework of the Swedish National Quality Registries [9]. In co-operation with the Public Health Agency of Sweden, mandatory surveillance data of COVID-19 are routinely reported. Written informed consent is not required, but patients may withdraw their data from the registry at any time. Available data in SIR include baseline demographics, comorbidities, variables included in the Simplified Acute Physiology Score (SAPS3) and variables on treatment given within the ICU. Data are recorded in raw format and transferred electronically to SIR after local validation. After central validation at SIR, incomplete or inconsistent (entries outside pre-specified limits) patient records are returned to the specific ICUs for correction before data are added to the master database. All Swedish citizens have a unique personal identity number making linkage possible to the Swedish Population Register, and thereby ascertain mortality data. It also enables to follow the care of a patient between different ICUs.

As of October 22 2020, 111,000 patients had been tested positive for SARS-CoV-2-RNA and 5921 deaths were registered due to COVID-19 in Sweden [10]. During the study period, a total of 23,664 COVID-19 patients had been hospitalized [11].

### Covariates and outcomes

Baseline characteristics were defined at the time of ICU admission. Physiological variables were recorded within one hour before until one hour after arrival to ICU. The worst set of data within this time interval was used. 90-day mortality was defined as death (all-cause mortality) within 90 days from first admission to ICU, respectively. The primary end-point was time to death after admission to ICU, and secondary end-point was 90-day mortality.

### Statistical analysis

Categorical variables are presented as number with percentage. Continuous variables are presented as median and interquartile range (IQR). Time to death was displayed using the Kaplan–Meier methodology, with comparison between curves using a log-rank test.

Association with mortality and a priori selected variables including patient sex, age, comorbidities (cardiac disease, chronic obstructive pulmonary disease (COPD)/asthma, morbid obesity (BMI > 40 kg/m^2^), hypertension, immune deficiency, chronic liver disease, chronic kidney disease, neuromuscular disease and malignancy (neoplasia spread beyond regional lymph nodes)), hospital level (local, county or tertiary) and admission month (March, April, May or June) were estimated using univariate and multivariable Cox regression models and expressed as hazard ratios (HR) with corresponding 95% confidence intervals. To avoid collinearity, age and comorbidity components were removed from SAPS3. In addition, in order to test the robustness of our findings, we performed a logistic regression model estimating factors associated with 90-day mortality. Results were expressed as odds ratios (OR) with corresponding 95% confidences intervals.

Data were analysed as complete cases. A *p* value of 0.05 was considered statistically significant. All data were analysed using Stata/SE 16 (StataCorp, Collage Station, TX, USA) and R 4.0.2 (R Core Team (2020). R: A language and environment for statistical computing. R Foundation for Statistical Computing, Vienna, Austria).

## Results

### Patients

From March 6 to June 30, 2020, a total of 2481 ICU patients with confirmed SARS-CoV-2 were reported to SIR. We excluded 127 patients who were admitted to ICU with a primary diagnosis not associated with COVID-19 (*n* = 64) and had a temporary (*n* = 62) Swedish personal identification number or invalid registration data (*n* = 1). In total, 2354 patients with laboratory-confirmed COVID-19 were included in the final analyses (see flow chart in Additional file 1). The median number of admissions per ICU was 25 (IQR 9–56, range 1–243). Of the total study cohort, only four patients (0.17%) were still in ICU at study end-point October 22, 2020.

### Demographics

Baseline characteristics are presented in Table 1. Of the included 2354 patient, 1722 (73.2%) were men. Median age was 61 (IQR 52–69) years for the entire patient cohort, 60 (IQR 50–70) and 61 (IQR 53–69) years for women and men, respectively. A majority of the patients (45%) were admitted during April. Median duration of symptoms before ICU admission was 10 (IQR 7–13) days. The majority of patients were admitted to tertiary (38.7%) or county (49.2%) hospitals. Of the 632 women included in the study, 20 (3.2%) were pregnant at ICU admission. Overall, 851 (36.2%) patients had no reported comorbidity at admission; 216 (34.2%) women and 635 (36.9%) men had no comorbidity. For the entire study cohort, the most common comorbidities were hypertension (40.6%) and diabetes (24.4%). Median SAPS 3 score at admission was 53 (IQR 47–60), corresponding to a median predicted risk of death of 22 (IQR 13–36)%. For women, median SAPS 3 score was 54 (IQR 46–60) and median predicted risk of death was 24 (IQR 14–36)%, and corresponding figures for men were 53 (46–60) and 22 (IQR 12–36)%.

**Table 1:** Baseline characteristics

Characteristics	No. (%)		
	All	Women	Men
No. (%)	2354	632 (26.8)	1722 (73.2)
Age, median (IQR), y	61 (52–69)	60 (50–70)	61(53–69)
*Age, interval, y*			
< 40	190 (8.1)	77 (12.2)	113 (6.6)
40–49	266 (11.3)	77 (12.2)	189 (11.0)
50–59	610 (25.9)	149 (23.6)	461 (26.8)
60–69	724 (30.8)	162 (25.6)	562 (32.6)
70–79	464 (19.7)	133 (21.0)	331 (19.2)
≥ 80	100 (4.2)	34 (5.4)	66 (3.8)
*Admission month*			
March	394 (16.7)	93 (14.7)	301 (17.5)
April	1064 (45.2)	270 (42.7)	794 (46.1)
May	557 (23.7)	162 (25.6)	395 (22.9)
June	339 (14.4)	107 (16.9)	232 (13.5)
*Location before ICU admission*			
Emergency department	537 (22.8)	123 (19.5)	414 (24.0)
Hospital floor	1815 (77.1)	508 (80.4)	1307 (75.9)
Time from symptom to ICU admission, median (IQR), d	10 (7–13)	9 (7–12)	10 (7–13)
*Hospital level*			
Tertiary	911 (38.7)	227 (35.9)	684 (39.7)
County	1158 (49.2)	311 (49.2)	847 (49.2)
Local	285 (12.1)	94 (14.9)	191 (11.1)
*Days at hospital before ICU admission, d*			
No. with data	2338	625	1713
Median (IQR)	1 (0–3)	2 (0–4)	1 (0–3)
Pregnancy		20 (3.2)	
*Comorbidities*			
None	851 (36.2)	216 (34.2)	635 (36.9)
One or more	1503 (63.8)	416 (65.8)	1087 (63.1)
Chronic hypertension	955 (40.6)	252 (39.9)	703 (40.8)
Chronic cardiac disease	286 (12.1)	47 (7.4)	239 (13.0)
COPD/Asthma	343 (14.6)	127 (20.1)	216 (12.5)
Immune deficiency	144 (6.1)	57 (9.0)	87 (5.1)
Chronic liver disease	19 (0.8)	8 (1.3)	11 (0.6)
Chronic kidney disease	113 (4.8)	25 (4.0)	88 (5.1)
Diabetes	574 (24.4)	143 (22.6)	431 (25.0)
Neuromuscular disease	34 (1.4)	10 (1.6)	24 (1.4)
Morbid obesity^a^	165 (7.0)	65 (10.3)	100 (5.8)
Malignancy^b^, No./total (%)	44/2338 (1.9)	14/625 (2.2)	30/1713 (1.8)
Vasopressor on admission, No./total (%)	96/2338 (4.1)	21/625 (3.4)	75/1713 (4.4)
Fever^c^, No./total (%)	977/2190 (44.6)	244/580 (42.1)	733/1600 (45.8)
*Glasgow coma scale*			
No. with data	10,690	252	808
Median (IQR)	15 (14–15)	15 (14–15)	15(14–15)[
*Systolic blood pressure, mmHg*			
No. with data	2181	582	1599
Median (IQR)	120 (100–140)	116.5 (100–134)	120 (103.5–140)
*Heart rate, beats/min*			
No. with data	2238	598	1640
Median (IQR)	95 (82–110)	95 (83–110)	95 (82–110)
*PaO*_*2*_*, mmHg*			
No. with data	2081	566	1515
Median (IQR)	9 (7.7–10.7)	9 (7.7–10.7)	9 (7.7–10.7)
*FiO*_*2*_*, %*			
No. with data	1583	429	1154
Median (IQR)	70 (60–85)	70 (60–89)	70 (58.5–85)
*PaO*_*2*_*/FiO*_*2*_* ratio*			
No. with data	1565	425	1140
Median (IQR)	13.1 (9.9–18.0)	12.9 (9.9–17.6)	13.2 (10.0–18.4)
*White blood cell count,* × *10*^*9*^*/L*			
No. with data	2134	570	1564
Median (IQR)	8.1 (6.0–10.9)	7.6 (5.7–10.9)	8.2 (6.2–10.9)
*pH*			
No. with data	2233	600	1633
Median (IQR)	7.45 (7.39–7.48)	7.44 (7.38–7.48)	7.45 (7.40–7.48)
*Creatinine, mg/dL*			
No. with data	2143	571	1572
Median (IQR)	0.8 (0.65–1.03)	0.63 (0.51–0.83)	0.85 (0.71–1.09)
*Bilirubin, mg/dL*			
No. with data	2057	546	1511
Median (IQR)	0.53 (0.35–0.7)	0.41 (0.29–0.58)	0.53 (0.41–0.76)
*SAPS 3 at admission, median (IQR)*			
No. with data	2338	625	1713
Median (IQR)	53 (47–60)	54 (48–60)	53 (46–60)
Predicted risk of death, median (IQR), %	0.22 (0.13–0.36)	0.24 (0.14–0.36)	0.22 (0.12–0.36)
30-day mortality	548 (23.3)	135 (21.4)	413 (24.0)
90-day mortality	634 (26.9)	148 (23.4)	486 (28.2)

*IQR* interquartile range, *y* years, *d* days, *ICU* intensive care unit, *COPD* chronic obstructive pulmonary disease, *PaO*_*2*_ arterial partial pressure of oxygen, *FiO*_*2*_ fraction of inspired oxygen, *SAPS* simplified acute physiology score

^a^Morbid obesity is defined as a body mass index above 40 kg/m^2^

^b^Malignancy is defined as neoplasia spread beyond regional lymph nodes

^c^Body temperature above 38 °C

### Process of care

Care provided in the ICU is presented in Table 2. Overall, 74.7% received invasive mechanical ventilation for a median total duration of 313 (IQR 186–534) hours. Corresponding figures for women were 70.3% with a duration of 266 (IQR 165–443) hours and for men 76.3% with a duration of 331 (191–571) hours. Renal replacement therapy and prone position were reported in 19.2% and 46.3% of the patients, respectively, and both treatments were more common in men than women. 23 patients received extra corporeal membrane oxygenation (ECMO). Median total length of ICU stay was 12 (IQR 5–22) days; 10 (IQR 4–18) days for women and 13 (IQR 6–24) days for men.

**Table 2:** Care provided in the ICU

Variable	No (%)		
	All (*n* = 2354)	Women (*n* = 632)	Men (*n* = 1722)
*Highest level of respiratory support*			
No. with data	2341	629	1712
Invasive mechanical ventilation	1749 (74.7)	442 (70.3)	1307 (76.3)
Non invasive mechanical ventilation	158 (6.7)	50 (7.9)	108 (6.3)
*Duration of invasive mechanical ventilation*			
No. with data	1746	441	1305
Median (IQR), h	313 (186–534)	266 (165–443)	331 (191–571)
Renal replacement therapy, No./total (%)	380/1984 (19.2)	72/539 (13.4)	308/1445 (21.3)
Prone position, No./total (%)	979/2119 (46.3)	231/582 (39.7)	748/1537 (48.6)
ECMO, No./total (%)	23/1368 (1.7)	7/353 (2.0)	16/1015 (1.6)
Tracheostomy, No./total (%)	654/2341 (27.9)	142/629 (22.6)	512/1712 (29.9)
One or more readmissions	314 (13.4)	74 (11.7)	240 (14.0)
ICU length of stay, median (IQR), d	12 (5–22)	10 (4–18)	13 (6–24)

*IQR* interquartile range, *ECMO* extracorporeal membrane oxygenation, *h* hours, *ICU* intensive care unit, *d* days

### Mortality

**Fig. 1:**
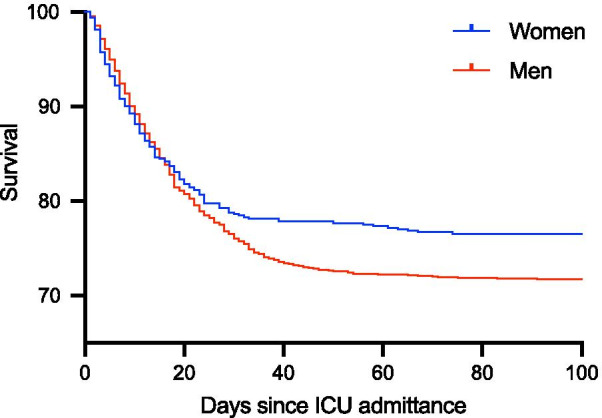
Time to death for men and women admitted to ICU. After 90 days, only 11 more deaths occurred. *p* = 0.016

Median follow-up time was 183 (IQR 158–199, range 114–230) days. In total, we observed a crude mortality at 30-days of 23.3%, in women 21.4% and in men 24.0%. Mortality at 90-days was 26.9 for the entire study cohort, 23.4% and 28.2% for women and men, respectively (Table 1). 90-days mortality per age group is depicted in Additional file 2. After 90 days until end of follow-up, only 11 deaths occurred. Overall time to death is displayed in Fig. 1 (*p* = 0.016).

**Fig. 2:**
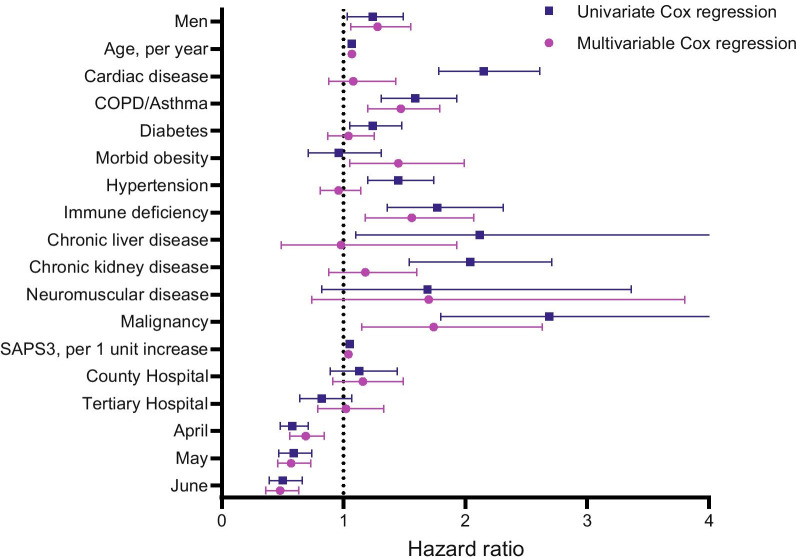
Univariate and multivariable Cox regression exploring 90-day mortality. Local hospital is considered as reference. March is considered as reference month

**Fig. 3:**
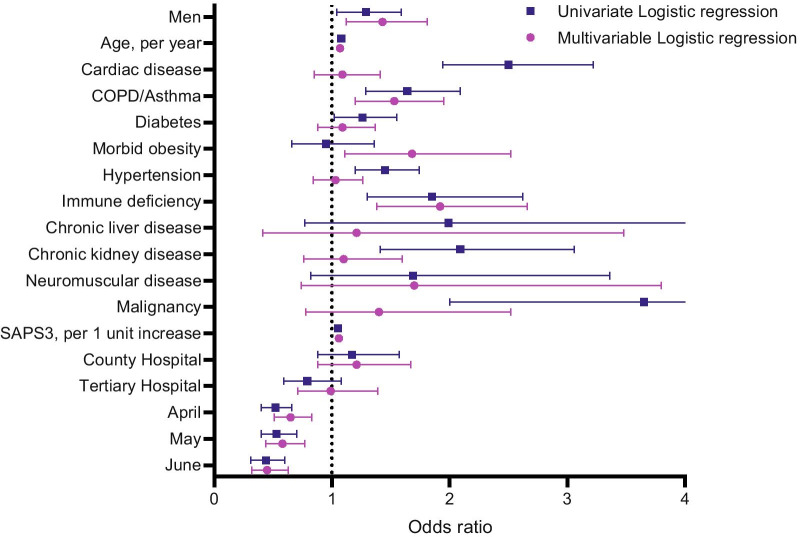
Univariate and multivariable logistic regression exploring 90-day mortality. Local hospital is considered as reference. March is considered as reference month

On univariate Cox regression analysis, male sex, age, cardiac disease, COPD/asthma, diabetes, hypertension, immune deficiency, chronic liver disease. chronic kidney disease, malignancy, SAPS3 (excluding age and comorbidity components) and admission month were significantly associated with mortality. On multivariable Cox regression analysis, male sex (HR 1.28, 95% CI 1.06–1.55) remained significantly associated with mortality even after adjustment for the above-mentioned covariates, also including morbid obesity and neuromuscular disease. In addition, age (HR 1.07, 95% CI 1.06–1.08 per year), COPD/asthma (HR 1.46, 95% CI 1.20–1.79), immune deficiency (HR 1.56, 95% CI 1.18–2.07), morbid obesity (HR 1.45, 95% CI 1.05–1.99), malignancy (HR 1.81, 95% CI 1.19—2.74), SAPS3 (HR 1.04, 95% CI 1.03–1.05 per unit increase) and admission month (HR 0.48, 95% CI 0.36–0.63, June vs. March) were also significantly associated with mortality (Fig. 2 and Additional file 3: Table S1). We could not demonstrate any statistically significant interaction between age and patient sex. To test the robustness of our results, we also performed a logistic regression model of 90-day mortality, with almost identical results (Fig. 3 and Additional file 3: Table S2).

## Discussion

This is, to the best of our knowledge, the first nationwide study with complete long-term follow-up in ICU-treated patient with COVID-19. All patients were followed at least 90 days after ICU admission, with a median follow-up time of 183 days. The fact that only 4/2354 patients were still at ICU at the study endpoint facilitates adequate calculations of long-term mortality after severe COVID-19. Crude 90-day mortality for the entire study cohort was 26.9%. Only 11 patients died after 90 days until end of follow-up. Despite adjustment for clinically relevant confounders male patients had an approximately 1.3 times higher risk of death compared to females. Furthermore, high age, COPD/asthma, immune deficiency, malignancy, morbid obesity, SAPS3 and admission month were associated with poor long-term outcome.

It is by now well established that men have more severe manifestations of COVID-19 including hospitalization, ICU admission and short-term mortality [4, 12]. Several studies demonstrate a male dominance in general ICU populations, where men constitute approximately 60% of the patients [13, 14]. This is lower compared to COVID-19 patients where men constitute an even larger proportion [15, 16], as also seen in the current study with 73% males. Complete understanding of mortality after ICU admission for COVID-19 is complicated by the fact that previous reports are inconsistent with regards to different follow-up times, but more important due to the fact that some included patients were still in ICU at study end-point. Thus, a large variety of mortality figures has been shown [17]. Furthermore, there is an obvious knowledge gap regarding long-term outcome also due to the novelty of the pandemic and in this context outcome beyond 90 days is fair to be considered as long-term outcome for COVID-19 patients. In the current study, all patients were followed at least 90-days, with a median follow-up time of 183 days.

The hazard ratio was 1.28 for men compared to women after adjustment for age, comorbidities, morbid obesity, SAPS3, hospital level and admission month. We could not find any other explanatory factor for this mortality difference than patient sex, although register-based studies obviously have its limitations. Only ICU patients were studied, and we have no data on patients hospitalized due to COVID-19, but not admitted to ICU. There is no previous published evidence suggesting differences between women and men regarding ICU admission *thresholds* for patients with COVID-19, nor differences in ICU treatment. It is, however, clear that men are worse after infection with SARS-Cov-2 [18, 19]. It is well known that male sex is associated with a poorer prognosis in a number of pulmonary clinical conditions [20]. The mechanisms causing this discrepancy is not fully understood and not the focus for this article, but it is hypothesised that it concerns either the effect of sex-hormones on inflammation [21] or X-chromosome linked molecules that are involved in inflammation, rendering women more prone to be protected against acute inflammation but more at risk for adverse outcome after chronic inflammation [20]. There is emerging evidence that sex differences in SARS-CoV-2 viral entry and immune responses against SARS-CoV-2 could contribute to these differences [22]. The field is rapidly evolving, and it has been suggested that men and women could benefit from different treatment approaches [23].

Besides the higher association with mortality for men compared to women in the present study, increasing age, COPD/asthma, immune deficiency, malignancy, morbid obesity, SAPS3 and admission month were associated with mortality. Age and various comorbid have been demonstrated as risk factors for poor outcome in ICU patients with COVID-19 [4]. Differences in the influence of comorbid conditions, as previously reported, could be partly explained by data availability, patient selection and various follow-up periods. For SAPS3, we excluded the age and comorbidity components in an attempt to avoid collinearity. Improved survival in patient admitted in the latter inclusion months compared to March is not surprising given the novelty of the pandemic and constant evolving evidence of optimal treatment strategies for critically ill patients with COVID-19 [24–26]. Furthermore, the higher mortality during the early months of the pandemic could also be an effect of patient volume per se*.*

Sweden has adopted a slightly different strategy during the pandemic than most other countries which has received some global interest. In contrast to country- or region-wide lockdowns, a combination of binding regulations and non-political nation-wide voluntary restrictions and quarantine recommendations has been applied. In Sweden, the first COVID-19 patient was admitted to an ICU on March 6, 2020 [27]. During the following months, there was a steady increase in ICU COVID-19 patients. In the beginning of July, there was a durable low incidence of new admissions, but in late October ICU admissions started to increase once again. Between Mars 6 and June 30, 2020, Sweden had a rapidly increasing death toll, predominately in the age group ≥ 80 where 3672 (63%) of the deaths occurred [10]. To date, Sweden has approximately 595 deaths/million inhabitants. This is more than other countries in Scandinavia, but similar to other European countries [1].

Complete follow-up was ascertained by linkage between SIR and national registries. In Sweden, all citizens have a personal identity number, except for a few newly arrived. SIR receive a list of all deceased or emigrated persons from the Swedish population registry once a week and the follow-up and date of death is included in the dataset for each ICU-admission as well as a code for any problems with the linkage*.* SIR includes high-resolution data on pre-ICU comorbid conditions, process of ICU care and long-term follow-up. Data in the register are validated in-house at all ICUs reporting to SIR, but also centrally by SIR before data are extracted. Furthermore, data are prospectively reported to SIR for quality-surveillance purposes and are therefore unbiased in relation to this study. Importantly, only four patients remained at ICU at study endpoint, thus making comprehensive analyses of mortality possible. All COVID-19 ICU patients in Sweden are included in the study, providing high generalizability to similar health-care systems.

As with all registry-based study designs, this study has limitations. Data on socioeconomic status and ethnicity may have added value to this study, but these data were unfortunately not available. The inclusion period was when the pandemic was at its first peak in Sweden. Even though there was a rapid increase in the number of ICU beds following early reports of the pandemic, ICU recourses were of course strained during this period. Data on ICU staffing during the study period were unavailable. Additionally, the worldwide cumulative knowledge concerning treatment of the disease increases during the course of the pandemic, and treatment algorithms are constantly evolving. For example, the introduction of high-dose low molecular weight heparins is not included in SIR, nor is dexamethasone [24, 25]. COVID-19 patients admitted to ICU from July 1, 2020, and onwards might have a different outcome than patients included in our study.

## Conclusion

In this nationwide study, we present complete long-term follow-up in ICU-treated patient with COVID-19. Critically ill men with COVID-19 are at higher risk of poor long-term outcome after ICU admission compared to their female counterparts. Differences remained after adjustment for relevant confounders including age, comorbidities, SAPS3 and admission month. The underlying explaining mechanisms for these differences are not fully understood and warrant further studies.

## Supplementary Information

**Additional file 1**. Flow chart.

**Additional file 2**. Depicts 90-days mortality per age group.

**Additional file 3**. Univariate and multivariable Cox and logistic regression analysis for overall mortality.

## Data Availability

Upon reasonable request and according to ethical approval.

## Abbreviations

COVID-19: Coronavirus disease 2019
ICU: Intensive care unit
SARS-CoV-2: Severe acute respiratory syndrome Coronavirus 2
SIR: Svenska intensivvårdregistret (Swedish Intensive Care Registry)
STROBE: Strengthening the Reporting of Observational Studies in Epidemiology
SAPS3: Simplified Acute Physiology Score 3
IQR: Interquartile range
COPD: Chronic obstructive pulmonary disease
HR: Hazard ratio
OR: Odds ratio

## Code availability

Upon reasonable request and according to ethical approval.

## References

[1] John Hopkins Coronavirus resource center [Internet]. Coronavirus, John Hopkins. [cited 2020 Nov 17]. https://coronavirus.jhu.edu/map.html.

[2] Armstrong RA, Kane AD, Cook TM (2020). Decreasing mortality rates in ICU during the COVID-19 pandemic. Anaesthesia.

[3] Quah P, Li A, Phua J (2020). Mortality rates of patients with COVID-19 in the intensive care unit: a systematic review of the emerging literature. Crit Care.

[4] Williamson EJ, Walker AJ, Bhaskaran K, Bacon S, Bates C, Morton CE (2020). Factors associated with COVID-19-related death using OpenSAFELY. Nature.

[5] Richardson S, Hirsch JS, Narasimhan M, Crawford JM, McGinn T, Davidson KW (2020). Presenting characteristics, comorbidities, and outcomes among 5700 patients hospitalized with COVID-19 in the New York City Area. JAMA.

[6] Kim L, Garg S, O’Halloran A, Whitaker M, Pham H, Anderson EJ (2019). Risk factors for intensive care unit admission and in-hospital mortality among hospitalized adults identified through the U.S. Coronavirus Disease 2019 (COVID-19)-Associated Hospitalization Surveillance Network (COVID-NET. Clin Infect Dis..

[7] Grasselli G, Zangrillo A, Zanella A, Antonelli M, Cabrini L, Castelli A (2020). Baseline characteristics and outcomes of 1591 patients infected With SARS-CoV-2 admitted to ICUs of the Lombardy Region. Italy JAMA.

[8] von Elm E, Altman DG, Egger M, Pocock SJ, Gøtzsche PC, Vandenbroucke JP (2007). The Strengthening the Reporting of Observational Studies in Epidemiology (STROBE) statement: guidelines for reporting observational studies. Lancet.

[9] Swedish National Quality Registries [Internet]. 2020; [cited 2020 Dec 10] Available from: https://kvalitetsregister.se/englishpages/findaregistry/allswedishqualityregistries.2028.html

[10] Folkhälsomyndigheten. Covid-19 in Sweden. [cited 2020 Nov 17]. https://www.folkhalsomyndigheten.se/smittskydd-beredskap/utbrott/aktuella-utbrott/covid-19/statistik-och-analyser/bekraftade-fall-i-sverige/.

[11] Socialstyrelsen. Socialstyrelsen [Internet]. Statistics concerning Covid-19. [cited 2020 Nov 17]. https://www.socialstyrelsen.se/statistik-och-data/statistik/statistik-om-covid-19/.

[12] Li LQ, Huang T, Wang YQ, Wang ZP, Liang Y, Huang TB (2020). COVID-19 patients’ clinical characteristics, discharge rate, and fatality rate of meta-analysis. J Med Virol.

[13] Valentin A, Jordan B, Lang T, Hiesmayr M, Metnitz PG (2003). Gender-related differences in intensive care: a multiple-center cohort study of therapeutic interventions and outcome in critically ill patients. Crit Care Med.

[14] Zettersten E, Jaderling G, Bell M, Larsson E (2019). Sex and gender aspects on intensive care. A cohort study. J Crit Care.

[15] Mitra AR, Fergusson NA, Lloyd-Smith E, Wormsbecker A, Foster D, Karpov A (2020). Baseline characteristics and outcomes of patients with COVID-19 admitted to intensive care units in Vancouver, Canada: a case series. CMAJ.

[16] Azoulay E, Fartoukh M, Darmon M, Géri G, Voiriot G, Dupont T (2020). Increased mortality in patients with severe SARS-CoV-2 infection admitted within seven days of disease onset. Intensive Care Med..

[17] Armstrong RA, Kane AD, Cook TM (2020). Outcomes from intensive care in patients with COVID-19: a systematic review and meta-analysis of observational studies. Anaesthesia.

[18] Li X, Xu S, Yu M, Wang K, Tao Y, Zhou Y (2020). Risk factors for severity and mortality in adult COVID-19 inpatients in Wuhan. J Allergy Clin Immunol.

[19] Palaiodimos L, Kokkinidis DG, Li W, Karamanis D, Ognibene J, Arora S (2020). Severe obesity, increasing age and male sex are independently associated with worse in-hospital outcomes, and higher in-hospital mortality, in a cohort of patients with COVID-19 in the Bronx. New York Metabolism.

[20] Casimir GJ, Lefèvre N, Corazza F, Duchateau J (2013). Sex and inflammation in respiratory diseases: a clinical viewpoint. Biol Sex Differ.

[21] Vom Steeg LG, Klein SL (2019). Sex and sex steroids impact influenza pathogenesis across the life course. Semin Immunopathol.

[22] Bunders MJ, Altfeld M (2020). Implications of sex differences in immunity for SARS-CoV-2 pathogenesis and design of therapeutic interventions. Immunity.

[23] Takahashi T, Ellingson MK, Wong P, Israelow B, Lucas C, Klein J (2020). Sex differences in immune responses that underlie COVID-19 disease outcomes. Nature.

[24] Miesbach W, Makris M (2020). COVID-19: coagulopathy, risk of thrombosis, and the rationale for anticoagulation. Clin Appl Thromb Hemost.

[25] Horby P, Lim WS, Emberson JR, Mafham M, Bell JL (2020). Dexamethasone in hospitalized patients with Covid-19—preliminary report. N Engl J Med..

[26] Beigel JH, Tomashek KM, Dodd LE, Mehta AK, Zingman BS, Kalil AC, et al. Remdesivir for the treatment of Covid-19—final report. N Engl J Med [Internet]. 2020 Oct 8 [cited 2020 Nov 18]; https://www.ncbi.nlm.nih.gov/pmc/articles/PMC7262788/. 10.1056/NEJMoa2007764 PMC7262788 32445440

[27] Svenska Intensivvårdsregistret [Internet]. https://www.icuregswe.org/en/about-sir/.

