# Supplementary figures and images for: Long-term outcome after intensive care for COVID-19: differences between men and women—a nationwide cohort study
Source: Crit Care. 2021 Feb 25;25:86. doi: 10.1186/s13054-021-03511-x (PMC7906087; doi:10.1186/s13054-021-03511-x)

**Figure 1. Flow chart of included patients**

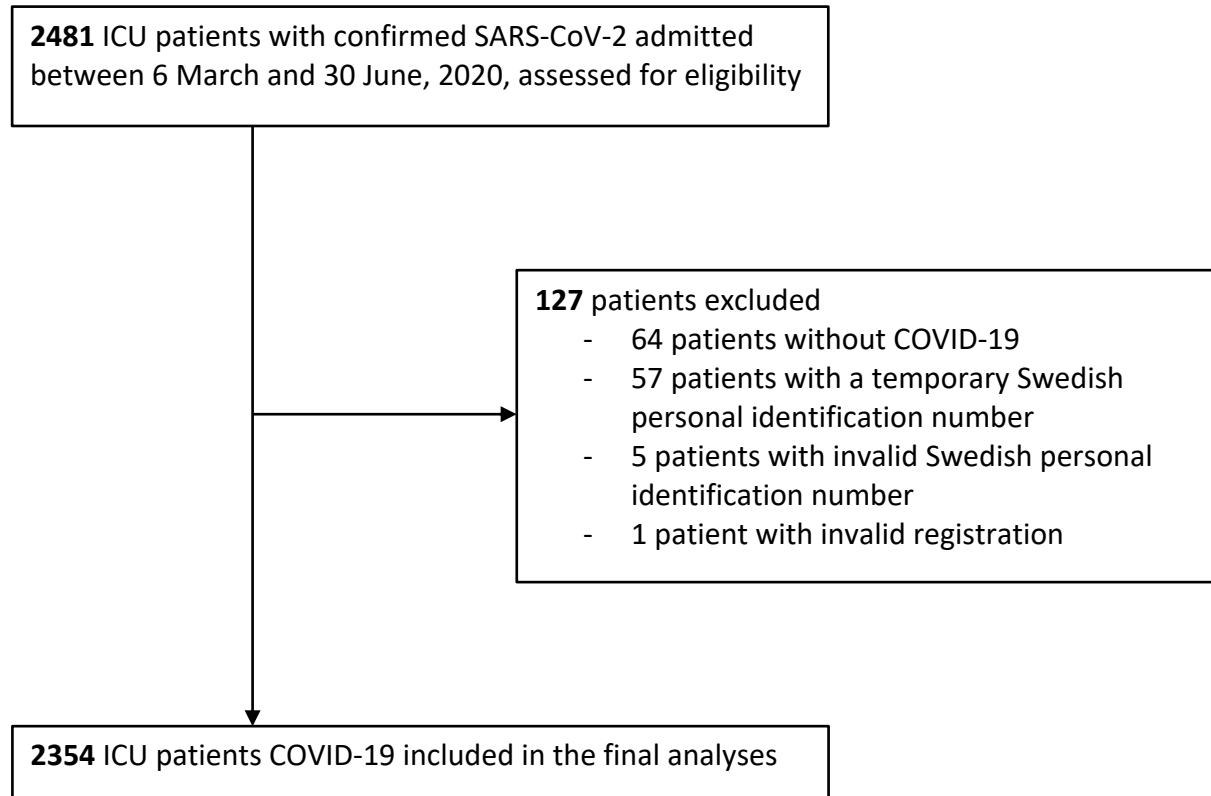

Supplement: Supplementary file 1 — Additional file 1. Flow chart. [file 13054_2021_3511_MOESM1_ESM.pdf]
