# Supplementary material for: Long-term outcome after intensive care for COVID-19: differences between men and women—a nationwide cohort study
Source: Crit Care. 2021 Feb 25;25:86. doi: 10.1186/s13054-021-03511-x (PMC7906087; doi:10.1186/s13054-021-03511-x)
Supplement: Supplementary file 3 — Additional file 3. Univariate and multivariable Cox and logistic regression analysis for overall mortality. [file 13054_2021_3511_MOESM3_ESM.docx]

**Table S1**

Univariate and multivariable Cox regression analysis for overall mortality

|  | Univariate | | Multivariable^a^ | |
| --- | --- | --- | --- | --- |
|  | HR (95% CI) | P value | HR (95% CI) | P value |
| Sex |  |  |  |  |
| Women | Reference |  | Reference |  |
| Men | 1.24 (1.03 - 1.49) | 0.021 | 1.28 (1.06 - 1.55) | 0.01 |
| Age, per year | 1.07 (1.06 - 1.08) | <0.001 | 1.07 (1.06 - 1.08) | <0.001 |
| Comorbidity |  |  |  |  |
| Cardiac disease | 2.15 (1.78 - 2.61) | <0.001 | 1.08 (0.88 - 1.34) | 0.44 |
| COPD/Asthma | 1.59 (1.31 - 1.93) | <0.001 | 1.47 (1.20 - 1.79) | <0.001 |
| Diabetes | 1.24 (1.05 - 1.48) | 0.013 | 1.04 (0.87 - 1.25) | 0.68 |
| Morbid obesity^b^ | 0.96 (0.71 - 1.31) | 0.80 | 1.45 (1.05 - 1.99) | 0.02 |
| Hypertension | 1.42 (1.21 - 1.65) | <0.001 | 0.96 (0.81 - 1.14) | 0.65 |
| Immune deficiency | 1.77 (1.36 - 2.31) | <0.001 | 1.56 (1.18 - 2.07) | 0.002 |
| Chronic liver disease | 2.12 (1.10 - 4.10) | 0.0248 | 0.98 (0.49 - 1.93) | 0.95 |
| Chronic kidney disease | 2.04 (1.54 - 2.71) | <0.001 | 1.18 (0.88 - 1.6) | 0.27 |
| Neuromuscular disease | 1.59 (0.92 - 2.75) | 0.098 | 1.42 (0.81 - 2.48) | 0.22 |
| Malignancy^c^ | 2.69 (1.8 - 4.01) | 0.011 | 1.74 (1.15 - 2.63) | 0.009 |
| SAPS3, per 1 unit increase^d^ | 1.05 (1.04 - 1.06) | <0.001 | 1.04 (1.03 - 1.05) | <0.001 |
| Hospital level |  |  |  |  |
| Local | Reference |  | Reference |  |
| County | 1.13 (0.89 - 1.44) | 0.32 | 1.16 (0.91 - 1.49) | 0.24 |
| Tertiary | 0.82 (0.64 - 1.07) | 0.14 | 1.02 (0.79 – 1.33) | 0.87 |
| Admission month |  |  |  |  |
| March | Reference |  | Reference |  |
| April | 0.58 (0.48 - 0.71) | <0.001 | 0.69 (0.56 - 0.84) | <0.001 |
| May | 0.59 (0.47 - 0.74) | <0.001 | 0.57 (0.46 - 0.73) | <0.001 |
| June | 0.50 (0.39 - 0.66) | <0.001 | 0.48 (0.36 - 0.63) | <0.001 |

Abbreviations: HR, Hazard Ratio; COPD, chronic obstructive pulmonary disease; SAPS, simplified acute physiology score

^a^2337patients included in the multivariable model

^b^Defined as BMI >40kg/m^2^

^c^Malignancy is defined as neoplasia spread beyond regional lymph nodes

^d^Recalculated after excluding age and comorbidities

**Table S2**

Univariate and multivariable logistic regression analysis for 90-day mortality

|  | Univariate | | Multivariable^a^ | |
| --- | --- | --- | --- | --- |
|  | OR (95% CI) | P value | OR (95% CI) | P value |
| Sex |  |  |  |  |
| Women | Reference |  | Reference |  |
| Men | 1.29 (1.04 - 1.59) | 0.02 | 1.43 (1.12 - 1.82) | 0.004 |
| Age, per year | 1.08 (1.07 - 1.09) | <0.001 | 1.08 (1.07 - 1.09) | <0.001 |
| Comorbidity |  |  |  |  |
| Cardiac disease | 2.50 (1.94 - 3.22) | <0.001 | 1.22 (0.90 - 1.63) | 0.19 |
| COPD/Asthma | 1.64 (1.29 - 2.09) | <0.001 | 1.45 (1.10 - 1.91) | 0.008 |
| Diabetes | 1.26 (1.02 - 1.55) | 0.03 | 1.06 (0.83 - 1.35) | 0.62 |
| Morbid obesity^b^ | 0.95 (0.66 - 1.36) | 0.79 | 1.68 (1.11 - 2.52) | 0.013 |
| Hypertension | 1.45 (1.2 - 1.74) | <0.001 | 0.89 (0.71 - 1.10) | 0.29 |
| Immune deficiency | 1.85 (1.3 - 2.62) | <0.001 | 1.64 (1.10 - 2.44) | 0.014 |
| Chronic liver disease | 1.99 (0.77 - 4.93) | 0.14 |  | 0.72 |
| Chronic kidney disease | 2.09 (1.41 - 3.06) | <0.001 | 1.22 (0.78 - 1.89) | 0.38 |
| Neuromuscular disease | 1.69 (0.82 - 3.36) | 0.14 | 1.70 (0.74 - 3.80) | 0.20 |
| Malignancy x | 3.65 (2.0 - 6.76) | <0.001 | 2.31 (1.19 – 4.57) | 0.01 |
| SAPS3, per 1 unit increase^c^ | 1.05 (1.04 - 1.07) | <0.001 | 1.05 (1.04 - 1.06) | <0.001 |
| Hospital level |  |  |  |  |
| Local | Reference |  | Reference |  |
| County | 1.17 (0.88 - 1.57) | 0.29 | 1.21 (0.88 - 1.67) | 0.25 |
| Tertiary | 0.79 (0.59 - 1.08) | 0.14 | 0.99 (0.71 - 1.39) | 0.95 |
| Admission month |  |  |  |  |
| March | Reference |  | Reference |  |
| April | 0.52 (0.40 - 0.66) | <0.001 | 0.62 (0.47 - 0.81) | <0.001 |
| May | 0.53 (0.40 - 0.70) | <0.001 | 0.47 (0.34 - 0.65) | <0.001 |
| June | 0.44 (0.31- 0.60) | <0.001 | 0.37 (0.26 - 0.54) | <0.001 |

Abbreviations: OR, Odds Ratio; COPD, chronic obstructive pulmonary disease; SAPS, simplified acute physiology score

^a^2338 patients included in the multivariable model

^b^Defined as BMI >40kg/m^2^

^c^Malignancy is defined as neoplasia spread beyond regional lymph nodes

^d^Recalculated after excluding age and comorbidities
